# Evaluation of Preclinical Assays to Investigate an Anthroposophic Pharmaceutical Process Applied to Mistletoe (*Viscum album* L.) Extracts

**DOI:** 10.1155/2014/620974

**Published:** 2014-05-04

**Authors:** Stephan Baumgartner, Heidi Flückiger, Matthias Kunz, Claudia Scherr, Konrad Urech

**Affiliations:** ^1^Hiscia Institute, Society for Cancer Research, Kirschweg 9, 4144 Arlesheim, Switzerland; ^2^Institute of Integrative Medicine, Gemeinschaftskrankenhaus, University of Witten-Herdecke, Gerhard-Kienle-Weg 4, 58313 Herdecke, Germany; ^3^Institute of Complementary Medicine, Inselspital, University of Bern, Imhoof-Pavillon, 3010 Bern, Switzerland

## Abstract

Extracts from European mistletoe (*Viscum album* L.) developed in anthroposophic medicine are based on specific pharmaceutical procedures to enhance remedy efficacy. One such anthroposophic pharmaceutical process was evaluated regarding effects on cancer cell toxicity *in vitro* and on colchicine tumor formation in *Lepidium sativum*. Anthroposophically processed *Viscum album* extract (APVAE) was produced by mixing winter and summer mistletoe extracts in the edge of a high-speed rotating disk and was compared with manually mixed *Viscum album* extract (VAE). The antiproliferative effect of VAE/APVAE was determined in five cell lines (NCI-H460, DU-145, HCC1143, MV3, and PA-TU-8902) by WST-1 assay *in vitro*; no difference was found between VAE and APVAE in any cell line tested (*P* > 0.14). Incidence of colchicine tumor formation was assessed by measurement of the root/shoot-ratio of seedlings of *Lepidium sativum* treated with colchicine as well as VAE, APVAE, or water. Colchicine tumor formation decreased after application of VAE (−5.4% compared to water, *P* < 0.001) and was even stronger by APVAE (−8.8% compared to water, *P* < 0.001). The high-speed mistletoe extract mixing process investigated thus did not influence toxicity against cancer cells but seemed to sustain morphostasis and to enhance resistance against external noxious influences leading to phenomenological malformations.

## 1. Introduction

Use of extracts from European mistletoe (*Viscum album* ssp.* album* L.) for cancer treatment is based on suggestions of Steiner [1], who founded anthroposophic medicine (AM) together with Wegman in the 1920s [2]. Subsequent preclinical research into mistletoe extracts revealed a multitude of highly interesting compounds (e.g., mistletoe lectins, viscotoxins, alkaloids, triterpenes, and oligo- and polysaccharides) with antitumoral (cytotoxic, antiangiogenic) as well as immunomodulating properties [3–5]. Clinical application increased overall survival time and improved quality of life of patients with various forms of cancer [6–8] and furthermore seemed to be safe [8, 9].

There is a variety of mistletoe extracts available, differing in mistletoe host trees and extraction methods as well as further pharmaceutical processing [10]. Mistletoe extracts used in anthroposophic medicine (Iscador, Helixor, abnobaVISCUM, Iscucin, and Isorel) rely on specific anthroposophic pharmaceutical procedures that were developed on the basis of suggestions of Steiner to enhance anticancer efficacy of mistletoe [11]. In general, extracts from mistletoe harvested in different seasons (summer and winter) are mixed together in a sophisticated, specifically designed apparatus.

From a pharmaceutical point of view, the question arises whether the anthroposophic pharmaceutical procedures applied indeed enhance anticancer efficacy as intended. This in turn leads to the following question: which methods are suited and applicable to study any such increase in remedy efficacy? In general, mechanism studies performed on the cellular level are considered to be the scientifically and ethically appropriate first step prior proceeding to animal or human experimentation.

Correspondingly, determination of the toxicity of differently processed mistletoe extracts in cancer cell lines can be seen as first step to study the pharmaceutical process in question. This approach is based on the basic assumption that cancer is a cell-based disease, caused by multiple mutations of normal cells resulting in malignant cells, eventually supported by nonmalignant cells in the environment [12, 13].

A complementary view of cancer is that this disease is primarily a phenomenon at the level of multicellular organization. According to this view, tumors form if the organism as a whole is too weak to control individual cell growth [14]. Lately, the tissue organization field theory (TOFT) was put forward to challenge the somatic mutation theory (SMT) of cancer [15]. Anthroposophic medicine also presumes tumors to be failures of morphostasis and that cancer is primarily a coordination problem of the different super- and subordinated organizational levels within an organism [16, 17]. Correspondingly, anthroposophic tumor therapy complements conventional antitumor therapy with special measures to reestablish superordinate control and regulation [18, 19].

Methods and tools of preclinical research in cancer depend on the basic underlying paradigm, that is, the basic concept and understanding of cancer, its causes, and its formation (e.g., SMT or TOFT). Approaches based on the somatic mutation theory firstly center on cell-based bioassays and consequently on cell-tissue interactions. Preclinical approaches in cancer research based on the morphogenetic paradigm are much less developed [14]. For principal reasons, these primarily center on cancer in nonhuman biological models as a disorder phenomenon of the general morphological structure of entire organisms and subsequently also tissue-cell interactions, preferably in three-dimensional* in vitro* models.

The aim of the present study was to compare two complementary preclinical approaches to investigate the effects of one particular anthroposophic pharmaceutical process designed to enhance mistletoe efficacy. In this process, extracts from European mistletoe harvested in summer and winter are mixed together in the edge of a high-speed rotating disk. First, possible alterations in cell-based toxicity were tested in a panel of five cancer cell lines. Second, we investigated the potential of a newly developed morphological bioassay in which correspondingly processed mistletoe extracts were examined regarding their potential to protect plants from the damaging, shape changing effects of colchicine (colchicine tumor formation in* Lepidium sativum* L.).

## 2. Materials and Methods

### 2.1. Mistletoe Extracts and Pharmaceutical Processing

To produce the constituents for the investigated mistletoe extracts, mistletoe plants (*Viscum album* L. ssp.* album*) growing on either apple trees (*Malus domestica* Borkh.) or pine trees (*Pinus sylvestris* L.) were harvested shortly before midsummer and end of the year, respectively. The one- to two-year-old leaves, the stems, the generative organs, and in the winter harvest the ripened berries were mechanically opened in a roll mill and extracted by fermentation with mistletoe derived lactobacillus in distilled water. After three-day fermentation, the extract was obtained by pressing and sterile filtration. The basic winter and summer mistletoe extracts possess a drug-extract ratio of 1 : 5 (extract of 200 mg mistletoe fresh plant in 1 mL fluid).

**Figure 1:**
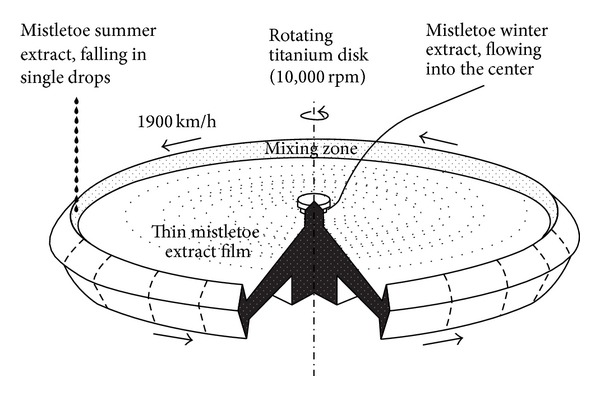
Schematic representation of the turning disc of the apparatus that has been used since autumn of 1996 at Hiscia Institute (Arlesheim, Switzerland) to produce anthroposophically processed* Viscum album* extract (APVAE). The winter mistletoe extract flows into the center of a 1 m diameter titanium disc rotating at 10,000 rpm and then spreads out horizontally. At the upturned disc edge (“mixing zone”), it combines with summer mistletoe extract dropping vertically from a height of 1 meter. Summer mistletoe extract enters the rotating disk not only at one position as schematically outlined above but also at 12 positions, equally spaced along the edge of the disk.

To produce anthroposophically processed* Viscum album* extract (APVAE), winter mistletoe extract is fed into the center of a 1 m diameter titanium disc rotating at 10,000 rpm. From here, it spreads out horizontally and combines subsequently with summer mistletoe extract dripping vertically from a height of 1 m into the upturned edge of the disc (Figure 1). The process runs continuously. After an average period of 20 seconds, the preparation leaves the disc edge. The disc is enclosed in an airtight stainless steel container floated with helium gas in order to avoid sonic boom, to reduce thermal friction loss, and to avoid oxidative reactions of the extract.

This specific blending process for winter and summer mistletoe extract was developed at the Hiscia Institute of the Society for Cancer Research (Arlesheim, Switzerland), with emphasis on realizing Steiner's original suggestions as precisely as possible [11, 20]. This process is being used for the production of Iscador.

As comparison sample relative to APVAE, we used a* Viscum album* extract (VAE) consisting of the same constituents as the APVAE sample (winter and summer mistletoe extracts of the same batch as used for production of APVAE). For this purpose, a container was successively filled with winter and summer mistletoe extracts that were mixed together homogenously by upending the container 10 times.

For the experiments, APVAE and VAE were sterile filtered immediately after mixing and filled into 1 mL glass ampoules under aseptic conditions for storage at 4°C. Two sorts of mistletoe extracts were investigated: extracts from mistletoe plants growing on the host tree apple (APVAE/VAE Mali) and extracts from mistletoe plants growing on the host tree pine (APVAE/VAE Pini).

### 2.2. Cell Culture

The human carcinoma cell lines HCC1143 (breast carcinoma), PA-TU-8902 (pancreas adenocarcinoma), DU-145 (prostate carcinoma), and NCI-H460 (lung carcinoma) were obtained from the Leibniz Institute DSMZ-German Collection of Microorganisms and Cell Cultures (Germany). MV3 cells (human metastatic melanoma) were kindly provided by Anka Dahl, University Hospital, Hamburg-Eppendorf (Germany). All cell lines except PA-TU-8902 were cultivated under standard cell culture conditions (37°C, 95% relative humidity, 5% CO_2_) in RPMI-1640 medium (Sigma-Aldrich, Buchs, Switzerland) supplemented with 10% heat-inactivated fetal bovine serum (FBS, Sigma-Aldrich, Switzerland), 2 mM L-Glutamine (Sigma-Aldrich, Switzerland), and Penicillin-Streptomycin (Sigma-Aldrich, Switzerland; Penicillin 10,000 units/mL and 10 mg/mL Streptomycin). Dulbecco's Modified Eagle's Medium (DMEM, Sigma-Aldrich, Switzerland) supplemented with the ingredients mentioned above was used to cultivate PA-TU-8902 cells. The cells were maintained in adherent, exponential growth in 25 cm^2^ culture flasks (TPP, Faust Laborbedarf AG, Switzerland) and cells from subconfluent monolayer were harvested by brief exposure to trypsin-EDTA solution (Sigma-Aldrich, Switzerland).

### 2.3. Cell Growth Assay

Cells were seeded at a density of 2500 cells in 90 *μ*L/well in a 96-well plate (TPP, Faust Laborbedarf AG, Switzerland) and incubated under standard conditions for 4 hours to attain adherence to the bottom. Thereafter, the cells were exposed during 48 hours to different concentrations of appropriate dilutions of* Viscum album* extract (VAE Mali, lot 1210/2338), anthroposophically processed* Viscum album* extract (APVAE Mali, lot 1210/2339), or blank medium (control) by adding a volume of 90 *μ*L/well resulting in a total volume of 180 *μ*L/well. After 2-day incubation, none of the cell lines had reached full confluence.

In this assay, only VAE/APVAE Mali was used due to the selective toxicity of the cancer cell lines used towards mistletoe lectins (VAE/APVAE Pini is almost void of mistletoe lectins). VAE Mali (lot 1210/2338) had a concentration of 10,400 ± 283 ng/mL mistletoe lectin, and APVAE Mali (lot 1210/2339) had a concentration of 10,350 ± 71 ng/mL. The mistletoe lectin content (ML I, ML II, and ML III) was determined by means of an ELISA with the aid of a combination of monoclonal antibodies specifically directed against the mistletoe lectins of mistletoes growing on deciduous trees [21].

Cell growth was assessed by using a colorimetric assay based on the cleavage of the tetrazolium salt WST-1 (Roche Diagnostica, Rotkreuz, Switzerland) to formazan by the mitochondrial dehydrogenase in viable cells [22]. For this purpose, after 48 h incubation of the cells, 20 *μ*L of WST-1 reagent was added and allowed to react for 4 h at 37°C. Quantification of the produced formazan was done by a multiwell spectrophotometer (Labsystem Multiskan RC, BioConcept AG, Allschwil, Switzerland) and absorbance was measured against a background control (cell free) at a wavelength of 450 nm and a reference wavelength of 690 nm. All substances were tested in triplicates and each experiment was repeated 8 times. Note that in this assay cell growth reflects the total mitochondrial reducing ability of the cell populations, which itself is strongly influenced by the corresponding proliferation rate (in a positive way) and by the occurrence of cell death (in a negative way).

Possible effects depending on the position of the samples within the 96-well plate were eliminated by systematic exchange of the sample positions. All manipulations by the experimenter were performed with coded (blinded) VAE and APVAE samples. VAE and APVAE samples were diluted to obtain the following final concentrations of the extracts: 800, 600, 400, 200, 100, 50, 25, and 12.5 *μ*g/mL. Furthermore, VAE/APVAE untreated cells were assessed. Each repetition of the experiments was done with freshly diluted extracts.

Viability of the cells was calculated by setting the OD values of the cell free background control to 0% and the OD values of the VAE/APVAE-untreated cells to 100%. ED50 was determined for each experiment by quadratic regression of viability versus concentration, including the concentrations with viability > 15% (12.5–200 *μ*g/mL for NCI-H460, DU-145, HCC1143, and MV3 and 12.5–800 *μ*g/mL for PA-TU-8902). Mean ED50 values ± standard deviation/error were calculated based on the eight ED50 values obtained in the eight independent experiments for each cell line. ED50 values for VAE and APVAE were compared using a *t*-test for independent samples. Calculations were carried out with Microsoft Excel for Mac 2011 (version 14.2.2) and Statistica 4 (Statsoft Inc., Tulsa, USA).

### 2.4. Morphological Colchicine Tumor Assay

Seedlings of* Lepidium sativum* L. (Ekkharthof, Lengwil, Switzerland) were cultivated in the dark at room temperature on chromatography paper (2043 A, Schleicher and Schuell, Dassel, Germany) in hanging LD-PE plastic bags (minigrip, Semadeni, Ostermundigen, Switzerland). Per bag, 16 sorted seeds were put on chromatography paper that had been soaked with 3 mL of fluid, namely, either (i) distilled water (Büchi, Fontavapor 250, Flawil, Switzerland), (ii) VAE Mali (lot 0404/4141) 2 mg/mL, (iii) APVAE Mali (lot 0404/4142) 2 mg/mL, (iv) VAE Pini (lot 0404/4143) 2 mg/mL, or (v) APVAE Pini (lot 0404/4144) 2 mg/mL. Mistletoe extracts were diluted 1 : 100 in distilled water (from 200 mg/mL to 2 mg/mL). Cultivation fluids were double coded and randomized. To all cultivation fluids, colchicine (Calbiochem, EMD Chemicals, San Diego, USA) was added at a fixed concentration within one experiment (see below).

Growth status was documented by photocopying the seedlings after 93 ± 4 h. The photocopies of the seedlings were put on a 12 × 12 inch graphics tablet (Summasketch III, Summagraphics, GTCO CalComp Inc., Scottsdale, USA) connected to an Apple Macintosh G3/233 Desktop computer. The shape of each seedling was digitized using special software (Tracking 0.2.6, Fritschy-Informatik, Zürich, Switzerland). Seedlings either partially invisible (hidden behind another seedling), without visible shoot or root, or growing off the chromatographic paper were not digitized. Tracking every seedling with the cursor of the graphics tablet resulted in a series of coordinates in the graphics tablet resolution (0.127 mm). In addition, the software calculated the true curve length of the seedling. Each seedling's length was divided into shoot and root length by marking the beginning and end of every measurement phase for both shoot and root. Hourly measurements of a cardboard template allowed a drift control of the graphics tablet in both *x*- and *y*-directions. Length measurements were carried out with coded samples.

A total number of seven independent experiments were carried out. Within one experiment, each experimental group comprised 20 bags, with each containing 16 seeds, amounting to 320 seeds per parameter per experiment or 11,200 seeds in total. Concentration of colchicine in the cultivation fluid was 17 *μ*g/mL for 1 experiment, 18 *μ*g/mL for 4 experiments, and 20 *μ*g/mL for 2 experiments.

**Figure 2:**
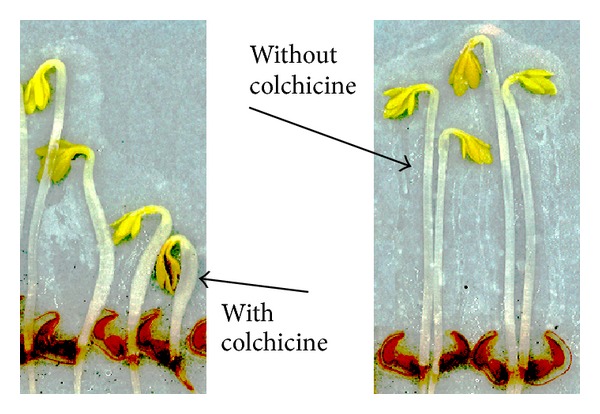
Colchicine (17 *μ*g/mL) induces shortening and thickening of the shoots when applied to* Lepidium sativum*.

Application of colchicine to* Lepidium sativum* led to dose-dependent shortening and thickening of the shoot (Figure 2) and to lengthening of the root. Hence, the ratio of root to shoot length increased due to colchicine. We therefore defined the ratio of root to shoot length as main outcome parameter for this morphological bioassay.

All data were analyzed with the statistics software Statistica 6 (Statsoft Inc., Tulsa, USA). Statistical evaluation was based on ANOVA (analysis of variance) procedures. Planned comparisons were evaluated with the LSD test only if the preceding global *F*-test was significant (*P* < 0.05) (protected Fisher's LSD). This procedure constitutes a good safeguard against type I as well as type II errors [23]. Statistical analysis was performed with data from *n* = 10,612 seedlings (data from 588 seedlings (5.25%) were missing, either due to failing germination or due to exclusion during length measurement according to the criteria defined above). Treatment parameters were decoded only after length measurement was accomplished.

## 3. Results

### 3.1. Antiproliferative Effects in Carcinoma Cell Lines

**Figure 3:**
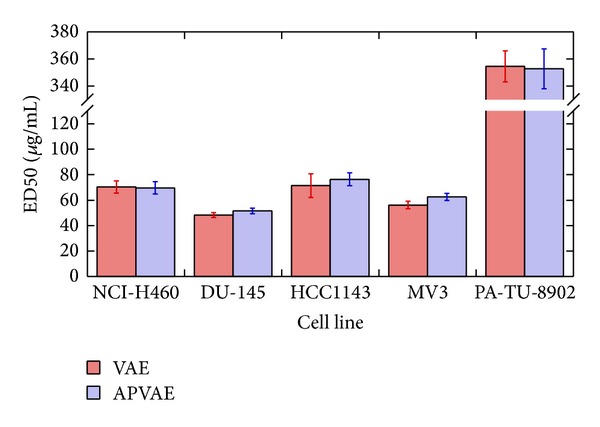
ED50 [*μ*g/mL] of* Viscum album* extract (VAE) Mali and anthroposophically processed* Viscum album* extract (APVAE) Mali in five different cell lines. Mean ± SE based on 8 independent experiments each. No significant differences (*P* < 0.05) were observed for any pairwise comparison between VAE and APVAE.

ED50 of* Viscum album* extract (VAE) Mali and anthroposophically processed* Viscum album* extract (APVAE) Mali was determined in five different cell lines (NCI-H460, DU-145, HCC1143, MV3, and PA-TU-8902), based on eight independent and coded (blinded) dose-response experiments each. ED50 of VAE Mali and APVAE Mali was statistically indistinguishable for all five cell lines (Table 1, Figure 3). In three cell lines (DU-145, HCC1143, and MV3), there was a trend for slightly elevated ED50 values for APVAE. In this assay, only VAE/APVAE Mali was investigated due to the selective toxicity of the cancer cell lines used towards mistletoe lectins (VAE/APVAE Pini is almost void of mistletoe lectins).

### 3.2. Morphological Bioassay

Experiments with colchicine-treated* Lepidium sativum* seedlings were analyzed with 2-way analysis of variance with the independent parameters treatment (*n* = 5: water control, VAE Mali, VAE Pini, APVAE Mali, and APVAE Pini) and experiment number (*n* = 7) and the dependent parameter root/shoot-ratio. All effects and interactions were highly significant (*P* < 0.0001, *F*-test; *n* = 10,612 in total).

The significant main effect for the independent parameter experiment number is due to the variations in absolute values of the outcome parameter root/shoot-ratio, which were most probably due to (i) the variations in colchicine concentration (17, 18, and 20 *μ*g/mL) and (ii) differences in room temperature between the seven independent experiments.

**Figure 4:**
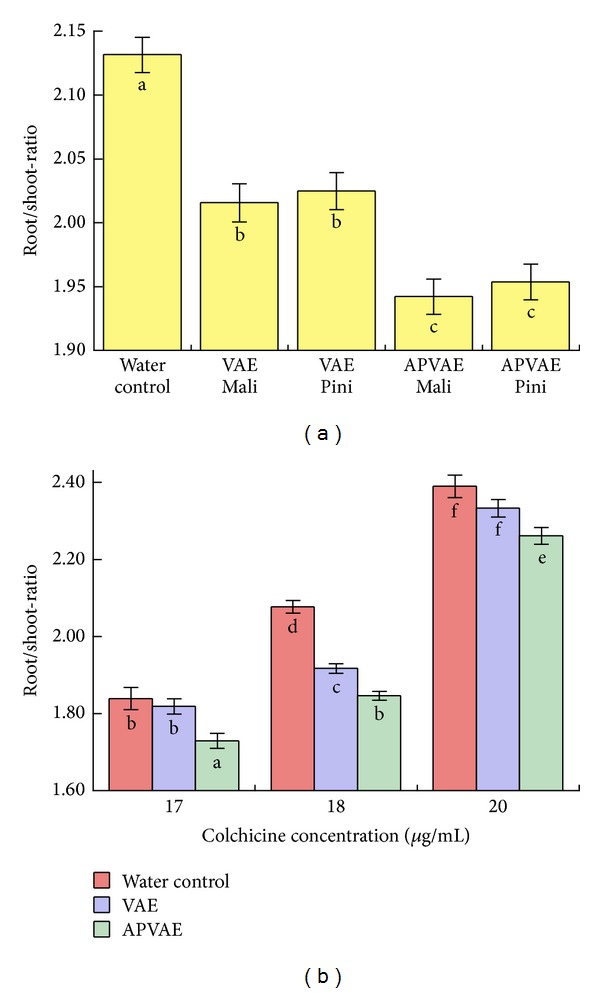
Root/shoot-elongation-ratio (mean ± SE) of colchicine-treated* Lepidium sativum*, with either water,* Viscum album* extract (VAE) Mali or Pini [2 mg/mL], or anthroposophically processed* Viscum album* extract (APVAE) Mali or Pini [2 mg/mL] added. (a) Average over all colchicine concentrations used. Parameters with different letters (a, b, and c) are statistically different (*P* < 0.001, LSD test); parameters with identical letters are statistically indistinguishable (*P* > 0.53, LSD test). (b) The same data as in (a) but differentiated according to the colchicine concentration used (17, 18, or 20 *μ*g/mL). Data from VAE Mali and VAE Pini as well as APVAE Mali and APVAE Pini were pooled. Parameters with different letters (a, b, c,…) are statistically different (*P* < 0.05, LSD test); parameters with identical letters are statistically indistinguishable (*P* > 0.05, LSD test).

The significant main effect for the independent parameter treatment reveals the impact of the mistletoe extracts on the root/shoot-ratio of* Lepidium sativum* seedlings (Figure 4(a)); the LSD test differentiates the effects of the treatments water control, VAE, and APVAE (*P* < 0.001 for all pairwise comparisons). No significant difference in the root/shoot-ratio of* Lepidium sativum* seedlings was observed regarding mistletoe host tree (Malus versus Pinus, cf. Figure 4(a)).

Application of colchicine leads to the formation of colchicine tumors on the shoots, which decreases shoot length and increases root length and consequently increases the root/shoot-ratio in elongation. Both VAE and APVAE Mali/Pini counteracted the effect of colchicine on* Lepidium sativum*. APVAE Mali and Pini preparations both seemed to exhibit a stronger impact compared to the VAE samples. On the average, VAE reduced the root/shoot-ratio by 5.4%, whilst APVAE reduced it by 8.8%, compared to the water control. The increase in root/shoot-ratio reduction by APVAE is about 60% relative to VAE and is highly significant (*P* < 0.001).

In order to assess the effects of the variations in colchicine concentration, a 2-way analysis of variance with the independent parameters treatment (*n* = 3: water control, VAE Mali/Pini, and APVAE Mali/Pini) and colchicine concentration (*n* = 3: 17, 18, and 20 *μ*g/mL) and the dependent parameter root/shoot-ratio was used. All effects and interactions were highly significant (*P* < 0.0001, *F*-test). The effects of VAE and APVAE were quite similar for the three colchicine concentrations, compared to the corresponding water control (Figure 4(b)). APVAE could be differentiated (*P* < 0.05) from the water control and VAE for all colchicine concentrations used; the effect of VAE was comparably weaker.

## 4. Discussion

The anthroposophic pharmaceutical process in question did not significantly alter the toxicity of* Viscum album* extracts in a panel of five carcinoma cell lines of different origin (pancreas adenocarcinoma, metastatic melanoma, and prostate, breast, and lung carcinoma). This result is in line with an earlier investigation of the same pharmaceutical process with two other cancer cell lines (Molt4 leukemia and Yoshida sarcoma cells) [24]. Viability reduction of carcinoma cell lines is most probably due to a specific compound found in aqueous mistletoe extracts, the mistletoe lectins [25]. In contrast, the Yoshida sarcoma cell line is specifically sensitive towards viscotoxins [26]. Thus, based on investigations in seven different cell lines, one may conclude that the anthroposophic pharmaceutical process studied does not seem to induce relevant changes in the biochemical properties of neither mistletoe lectins nor viscotoxins, though modifications of the complex molecules (60 kD and 5 kD, resp.) are conceivable due to the large sheering forces applied in the high-speed blending machine. In the present as well as an earlier investigation [24], concentration of lectins and viscotoxins was found unchanged after applying the anthroposophic pharmaceutical process in question.

In contrast, comparably large and highly significant differences between anthroposophically processed and unprocessed mistletoe extracts (APVAE versus VAE) were observed in the morphological bioassay with* Lepidium sativum*. The formation of the so-called colchicine tumors was reduced by application of VAE, and this reduction of tumor formation was enforced by APVAE, as depicted by the changes in the root/shoot-ratio being correlated to the formation of colchicine tumors. Furthermore, this decrease in tumor formation was comparable for extracts from mistletoe growing on the host trees apple and pine (VAE/APVAE Mali and Pini, resp.). VAE/APVAE Pini has only very low concentrations of mistletoe lectins, and concentration and composition of viscotoxins differ to a large extent between apple and pine mistletoe extracts [27]. Thus, neither mistletoe lectins nor viscotoxins can be the cause for the observed reduction in colchicine tumor formation.

In plants, colchicine inhibits microtubule assembly by binding to the dimeric subunit of the microtubule, tubulin [28]. This leads to reduction of cell polarity and—in dividing cells—also to polyploidy [29, 30], with the consequent formation of colchicine tumors, consisting of malformed cells or cell assemblies [31]. An interference of some compound(s) present in mistletoe extracts with the tubulin-colchicine reaction is in principle conceivable; we currently have no hypothesis which substance(s) might be responsible for any such interference, however. According to the results obtained and discussed above, mistletoe lectins and viscotoxins most probably have to be excluded as possible candidates for such interference.

The results of the present study are in line with an earlier investigation of colchicine tumor formation in* Triticum aestivum* shoots treated with VAE and APVAE Mali: an analogous reduction of tumor development through application of VAE Mali and an additional decrease of tumor incidence through application of APVAE Mali were observed [32]. Similarly, crown-gall-tumor formation in* Kalanchoe daigremontiana* induced by* Agrobacterium tumefaciens* was reduced through application of VAE Mali; this decrease was further enforced after application of APVAE Mali [24]. Furthermore, damage induced by ultraviolet radiation (UV) on* Triticum aestivum* shoots as well as* Sinapis alba* shoots could be alleviated after application of VAE and APVAE Mali, but with stronger effects of APVAE compared to VAE [32].

Summarizing, effects of the anthroposophic pharmaceutical process in question were observed in five different whole system bioassays (colchicine tumor formation in* Lepidium sativum*, colchicine tumor formation in* Triticum aestivum*, crown-gall-tumor formation in* Kalanchoe daigremontiana*, UV damage in* Triticum aestivum*, and UV damage in* Sinapis alba*). In all five bioassays, anthroposophically processed* Viscum album* extracts (APVAE) induced a stronger morphostatic protection effect than unprocessed* Viscum album* extract (VAE). In contrast, no unambiguous effects of the investigated anthroposophic pharmaceutical process were observed regarding toxicity of* Viscum album* extracts in seven cancer cell lines (NCI-H460, DU-145, HCC1143, MV3, PA-TU-8902, Molt4, and Yoshida) of different origins (pancreas adenocarcinoma, metastatic melanoma, prostate, breast, and lung carcinoma, leukemia, and sarcoma).

This means that the high-speed blending process of summer and winter mistletoe extracts seems to induce some specific properties in* Viscum album* extracts that support morphostasis, that is, that help entire organisms to maintain their organization and form when threatened by noxious external factors. In contrast, no clear effect of the blending process was observed on lectin-  and viscotoxin-based toxicity against cancer cell lines. Thus, it seems that preclinical methods derivable from the basic structure of the tissue organization field theory (TOFT) of cancer are more apt to investigate the anthroposophic pharmaceutical process in question, compared to preclinical methods derived from the somatic mutation theory (SMT) of cancer.

Anthroposophically processed* Viscum album* extracts from the host tree pine (APVAE Pini) led to a stabilization of the DNA of amniotic fluid cells* in vitro* [33]. Furthermore, APVAE Mali led to an improvement of DNA repair in gamma-ray or 4-hydroxycyclophosphamide damaged peripheral blood mononuclear cells (PBMC)* in vitro* [34]. A similar protection effect of APVAE Pini in PBMC poisoned with 4-hydroxycyclophosphamide was observed in a follow-up* in vitro* study, alongside the absence of any such protection effect in malignant Jurkat cells [35]. None of these three investigations was able to identify possible compounds of the mistletoe extracts that might be responsible for this DNA stabilizing effect.

In animal trials, anthroposophically processed* Viscum album* extracts exerted protective effects against carcinogenic compounds (N-[4-(5-nitro-2-furyl)-2-thiazolyl]-formamide, 20-methylcholanthrene) in trials with rats and mice [36, 37]. Furthermore, treatment with APVAE Mali resulted in a faster recovery from radiation- or cyclophosphamide-induced leukopenia in mice [38].

The results of the abovementioned preclinical investigations are in line with observations made in clinical trials. Kovacs et al. observed that DNA repair in lymphocytes of breast cancer patients could be substantially improved after subcutaneous injections of APVAE Mali [39]. Furthermore, there are several clinical investigations that documented a reduction of side effects of conventional antitumor therapy by simultaneous APVAE therapy, without reducing the former's efficacy [40–43].

Taken together, these preclinical and clinical observations support the hypothesis that the application of APVAE may help organisms (cells, plants, animals, and humans) in their continuing quest for maintenance of organization and form, especially when endangered by external noxious influences or by endogenous tumor formation. In addition, based on the results of the present and earlier investigations [24, 32], we put forward the hypothesis that part of the effects of APVAE is due to the specific anthroposophic pharmaceutical processing applied.

## 5. Conclusions

Summarizing, preclinical and clinical observations seem to suggest that anthroposophically processed mistletoe extracts may support entire organisms in general (cells, plants, animals, and humans) in their maintenance of organization and form, when endangered by endogenous tumor formation or externally applied noxious influences. We raise the hypothesis that part of the observed morphostatic effects of anthroposophically processed* Viscum album* extracts is due to the specific anthroposophic pharmaceutical processing applied.

Furthermore, we argue that remedies and pharmaceutical procedures of anthroposophic medicine and homeopathy, as well as from other branches of CAM in general (e.g., European or Chinese phytotherapy), should be investigated by preclinical assays that are adapted to the theoretical context from which they were developed. Therefore, we see a need to further develop preclinical approaches in cancer research based on the morphogenetic paradigm, centering on cancer in nonhuman biological models as a disorder phenomenon of the general morphological structure of entire organisms and subsequently also tissue-cell interactions, preferably in three-dimensional* in vitro* models.

## Figures and Tables

**Table 1:** Comparison of ED50 [*μ*g/mL] values of *Viscum album* extract (VAE) Mali and anthroposophically processed *Viscum album* extract (APVAE) Mali in five different cell lines. Mean ± SD based on 8 independent experiments each. 4th column: APVAE ED50 values expressed relative to VAE data set to 100%. 5th column: *t*-test for independent samples.

Cell line	ED50 VAE Mali	ED50 APVAE Mali	ED50 APVAE Mali	*P* (*t*-test)
	[mean ± SD]	[mean ± SD]	[%]	
NCI-H460	70 ± 13 µg/mL	70 ± 14 µg/mL	99.0%	0.915
DU-145	48 ± 5 µg/mL	52 ± 6 µg/mL	106.7%	0.277
HCC1143	71 ± 26 µg/mL	76 ± 14 µg/mL	106.9%	0.647
MV3	56 ± 8 µg/mL	63 ± 8 µg/mL	111.3%	0.141
PA-TU-8902	355 ± 32 µg/mL	353 ± 42 µg/mL	99.5%	0.922
